# Investigation of COVID-19 Outbreak among Wildland Firefighters during Wildfire Response, Colorado, USA, 2020

**DOI:** 10.3201/eid2808.220310

**Published:** 2022-08

**Authors:** Amanda Reiff Metz, Matthew Bauer, Chelsey Epperly, Ginger Stringer, Kristen E. Marshall, Lindsey Martin Webb, Molly Hetherington-Rauth, Shannon R. Matzinger, Sarah Elizabeth Totten, Emily A. Travanty, Kristen M. Good, Alexis Burakoff

**Affiliations:** Colorado Department of Public Health and Environment, Denver, Colorado, USA (A.R. Metz, C. Epperly, G. Stringer, K.E. Marshall, L.M. Webb, M. Hetherington-Rauth, S.R. Matzinger, S.E. Totten, E.A. Travanty, K.M. Good, A. Burakoff);; Larimer County Department of Health and Environment, Fort Collins, Colorado, USA (M. Bauer);; Centers for Disease Control and Prevention, Atlanta, Georgia, USA (K.E. Marshall)

**Keywords:** COVID-19, SARS-CoV-2, public health, disease outbreaks, whole-genome sequencing, social network analysis, phylogeny, firefighters, Colorado, physical distancing, fires, wildfire, coronavirus disease, severe acute respiratory syndrome coronavirus 2, viruses, respiratory infections, zoonoses

## Abstract

A COVID-19 outbreak occurred among Cameron Peak Fire responders in Colorado, USA, during August 2020–January 2021. The Cameron Peak Fire was the largest recorded wildfire in Colorado history, lasting August–December 2020. At least 6,123 responders were involved, including 1,260 firefighters in 63 crews who mobilized to the fire camps. A total of 79 COVID-19 cases were identified among responders, and 273 close contacts were quarantined. State and local public health investigated the outbreak and coordinated with wildfire management teams to prevent disease spread. We performed whole-genome sequencing and applied social network analysis to visualize clusters and transmission dynamics. Phylogenetic analysis identified 8 lineages among sequenced specimens, implying multiple introductions. Social network analysis identified spread between and within crews. Strategies such as implementing symptom screening and testing of arriving responders, educating responders about overlapping symptoms of smoke inhalation and COVID-19, improving physical distancing of crews, and encouraging vaccinations are recommended.

The Cameron Peak Fire in Colorado, USA, began on August 13, 2020. Because of the magnitude of this wildfire, the response was coordinated by various Incident Management Teams (IMT); wildfire responders included Colorado wildland firefighter crews as well as crews from around the country deployed to Colorado for the response. On August 25, 2020, the Larimer County Department of Health and Environment (LCDHE) and the Colorado Department of Public Health and Environment (CDPHE) received notification of a wildland firefighter responding to the Cameron Peak Fire who tested positive for SARS-CoV-2, the virus that causes COVID-19. This firefighter initially reported difficulty breathing and was transported to the local emergency department, then released. The next day, he was admitted to the hospital for continuing symptoms and tested positive for SARS-CoV-2 by reverse transcription PCR (RT-PCR). LCDHE, in partnership with the IMT, began contact tracing on the basis of the Centers for Disease Control and Prevention (CDC) definition of someone who was within 6 feet of an infected person for a cumulative 15 minutes or more over a 24-hour period (*1*). Two persons working on the same crew and 5 additional responders at the camp were identified as close contacts and quarantined. During contact interviews, it was reported that 2 crew members of the index case-patient were experiencing cough and headaches; both subsequently tested positive for SARS-CoV-2. An outbreak was declared and reported on September 2, 2020.

Wildfire response personnel operating across the state were in contact with CDPHE throughout the wildfire season regarding COVID-19 prevention and response plans. In July 2020, before the Cameron Peak Fire, CDPHE released public guidance documents addressing best practices for mitigating COVID-19 risks at wildfire camps (*2*). This document supplemented best practice guidance available from other sources such as CDC (*3*), United States Forest Service (USFS) (*4*), the Fire Management Board (*5*), and United States Department of the Interior (*6*). At the time that the Cameron Peak Fire started, a CDPHE occupational health epidemiologist regularly attended a morning safety briefing call organized by the USFS, in which incident management team representatives from all active fires in Colorado called in with updates on safety concerns including COVID-19.

## Methods

### Case Investigations

LCDHE and the Cameron Peak IMT collaborated to conduct case investigations and contact tracing activities. An outbreak case was defined as confirmed or probable COVID-19 (determined using the Council of State and Territorial Epidemiologists’ 2020 Interim COVID-19 Case Definition) (*7*) in a responder who was onsite at the Cameron Peak Fire within 14 days of symptom onset or positive test. Close contacts were identified on the basis of the CDC definition and quarantined. CDPHE and local hospital laboratories conducted SARS-CoV-2 RT-PCR testing using various platforms.

Outbreak response consultation calls among CDPHE, LCDHE, and IMT were held to provide recommendations for isolation of cases, quarantine of close contacts, and prevention practices such as improving physical distancing. CDPHE’s Rapid Response Team hosted a testing event for Cameron Peak Fire responders before the first positive case was identified; surveillance and outbreak screening testing was offered to all Cameron Peak Fire responders starting August 24. Once the outbreak was identified, widespread testing was conducted 11 more times during August 26–October 25, 2020. After fire, the USFS conducted a Facilitated Learning Analysis to identify lessons learned from the outbreak response (*8*).

### Whole-Genome Sequencing

CDPHE performed tiled amplicon whole-genome sequencing (WGS) on 40 (51%) available specimens from wildfire responders (Appendix); the remainder of the specimens were unavailable for sequencing because they were not sent to the CDPHE laboratory. We assembled sequencing data by using the Monroe workflow and CDPHE’s publicly available Nanopore data workflow (https://github.com/CDPHE).

**Figure 1:**
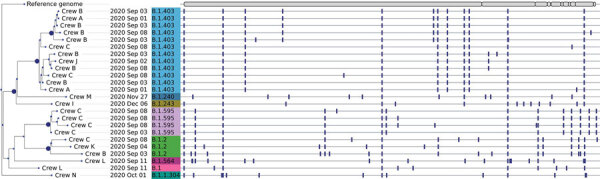
Phylogenetic tree of SARS-CoV-2 consensus whole-genome sequences from 24 of 42 positive specimens from Cameron Peak firefighters available at the Colorado State Public Health Lab with >89% genome coverage. Nodes with at least 95% ultrafast bootstrap support are labeled. Firefighter crew, sample collection date, and lineage are displayed at the tips. A visualization of the reference genome is depicted at the top of the phylogeny. Vertical bars shown across each consensus sequence indicate positions of nucleotide changes relative to the reference genome. High-quality consensus sequences were defined as sequences with >89% genome coverage (10× sequence coverage depth for Illumina [https://www.illumina.com] and 20× for Oxford Nanopore [https://nanoporetech.com]) and minimum base quality of 20. Prior to phylogenetic inference, consensus sequences were aligned to the reference genome (Genbank accession no. NC_045512.2), and insertions were removed so that all sequences were 29,903 nt in length. Phylogenetic inference of the consensus sequences was performed using IQTree version 2.0.3 (http://www.iqtree.org) with 1,000 ultrafast bootstrap replicates and phylogenetic tree visualization was performed using the python module ete3 version 3.1.2 (https://pypi.org/project/ete3). Pangolin v.2.4.2^5^ (*9*) and Nextstrain’s Nextclade tools (*10*) were used to assign lineage and clade designations to each assembled genome.

**Figure 2:**
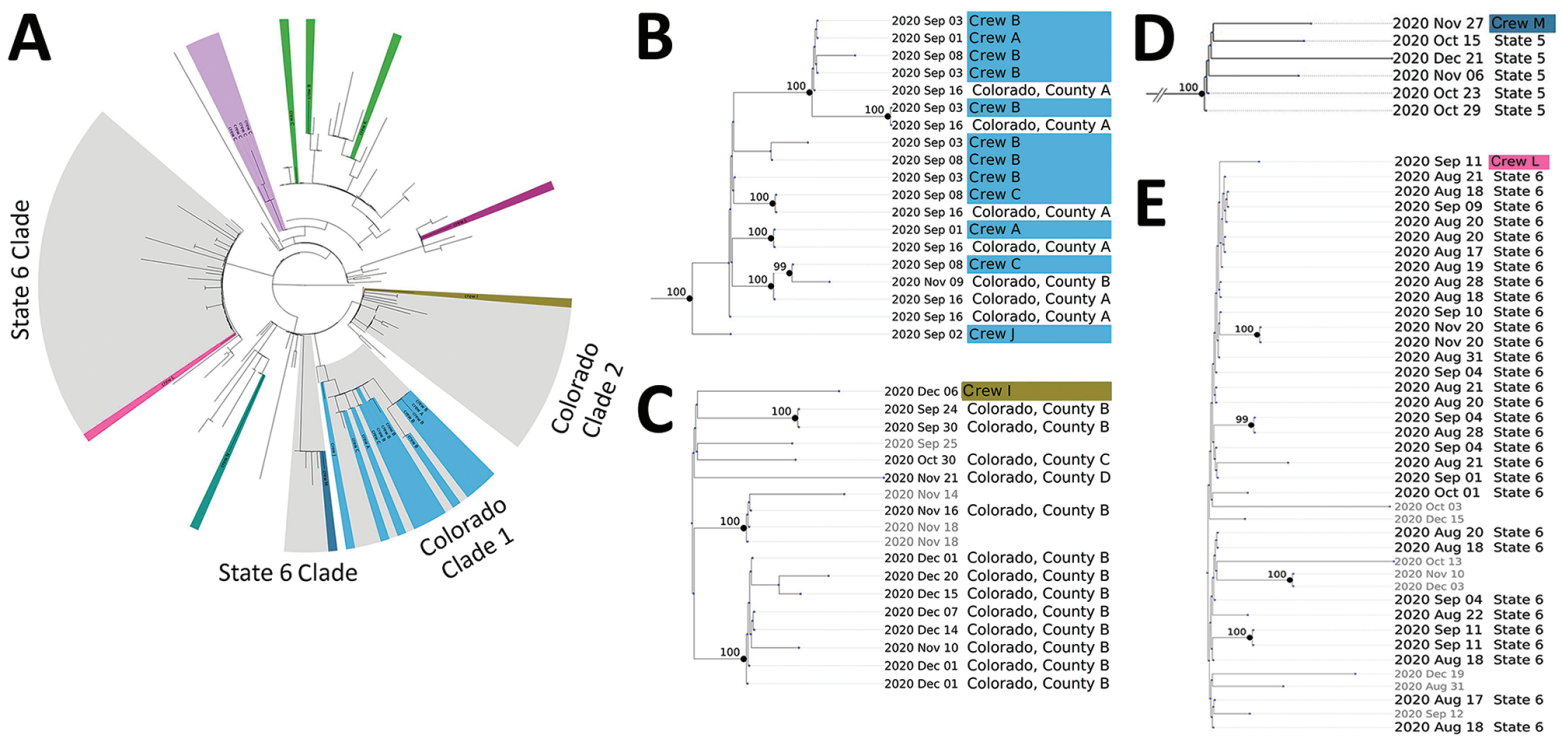
Contextual phylogenetic tree and enlarged clades showing genetic relatedness of the Cameron Peak firefighter sequences to sequences of SARS-CoV-2 collected within the United States during September–December 2020. A) Full contextual tree constructed using 754 contextual sequences subsampled from GISAID (https://www.gisaid.org) plus 24 Cameron Peak firefighter consensus sequences. The phylogeny has been pruned to display 164 contextual sequences and Cameron Peak firefighter sequences. Cameron Peak sequences are highlighted in color according to their lineage assignment. Clades highlighted in gray represent potential community and interstate transmission events. Cameron Peak sequences assigned to lineage B.1.2 (green) do not cluster together on the contextual phylogeny to form a monophyletic group, suggesting that they are genetically divergent from one another and likely do not represent a single transmission event, despite belonging to the same lineage. Mutation differences among these sequences are shown in detail in Figure 1. B) Colorado clade 1. Twelve Cameron Peak firefighters formed a monophyletic group with sequences from 2 Colorado counties. C) Colorado clade 2. A single Cameron Peak firefighter sequence formed a clade with sequences collected from 3 Colorado counties and additional sequences collected from outside of Colorado (not labeled). Low support values for this clade may be expected because of low sequence diversity. D) State 5 clade. The Cameron Peak firefighter sequence formed a monophyletic clade with sequences collected from his or her state of deployment (State 5). E) State 6 clade. The Cameron Peak firefighter sequence formed a clade with sequences collected from his or her state of deployment (state 6) and additional sequences collected from outside of Colorado and not from his or her state of deployment (not labeled).  Low support values for this clade may be caused by low sequence diversity. For panels B–E, all sequences within a clade are assigned the same lineage. Collection dates are labeled for all tips. Cameron Peak firefighter sequences are highlighted according to their lineage and labeled with crew. Nodes with at least 95% ultrafast bootstrap support values are labeled. Additional information is available in the Appendix.

Of the specimens available for WGS, 24 resulted in sequence determination; we used those sequences to construct a focal phylogenetic tree of the Cameron Peak Fire outbreak (Figure 1). In addition, to investigate the potential for multistate lineage introduction or community transmission, we constructed a contextual phylogenetic tree by using the 24 whole-genome sequences of the Cameron Peak Fire specimens and additional whole-genome sequences that were either publicly available or additionally sequenced at the CDPHE State Public Health Laboratory (Figure 2; Appendix).

### Social Network Analysis

We conducted social network analysis of all SARS-CoV-2–positive responders by using R Studio version 1.2.5033 (https://www.rstudio.com) and Gephi Graph Visualization and Manipulation software version 0.9.2 (https://gephi.org). We applied this analysis to WGS results to visualize clusters and transmission dynamics among Cameron Peak Fire crews (*11*). We assumed epidemiologic links of exposure between responders belonging to the same crew for network construction. Data showing potential exposure outside of crew assignments (i.e., socializing with members of other crews) were not available. This activity was reviewed by CDC and was conducted consistent with applicable federal law and CDC policy (45 C.F.R. part 46, 21 C.F.R. part 56; 42 U.S.C. Sect. 241(d); 5 U.S.C. Sect. 552a; 44 U.S.C. Sect. 3501 et seq).

## Results

**Figure 3:**
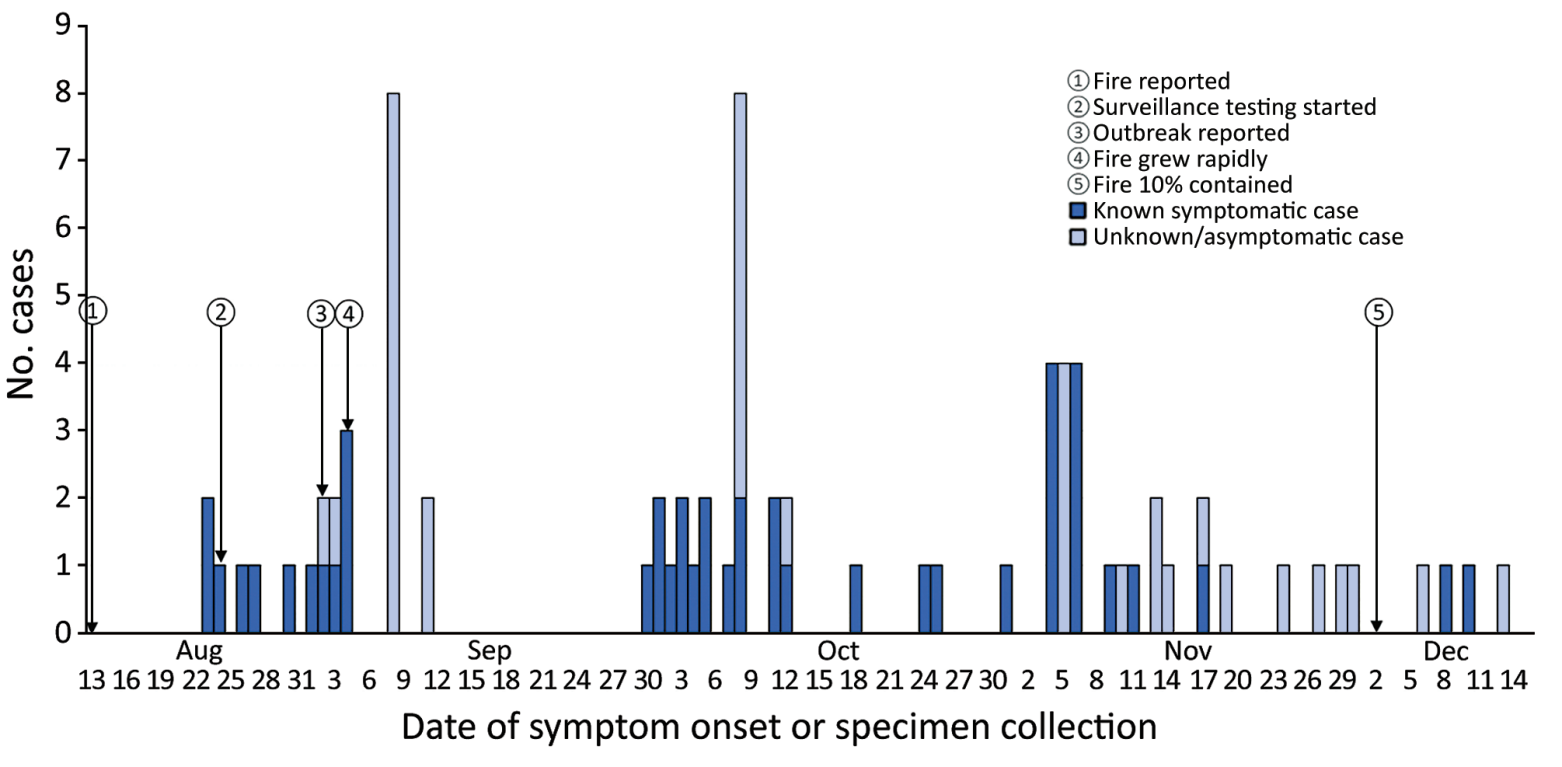
Timeline of COVID-19 outbreak among 79 firefighters during the Cameron Peak Fire, Colorado, USA, August–December 2020

The outbreak among wildfire responders occurred during August 25, 2020–January 8, 2021. (In Colorado, an outbreak is considered resolved 28 days after symptom onset of the last case.) A total of 6,123 responders were involved in the response. We identified 79 cases (78 confirmed and 1 probable); 73 of these were confirmed to be firefighters from 1 of the 63 crews, for an attack rate of 5.8% among 1,260 firefighters who were deployed full-time to the incident (Figure 3). The remainder of responder case-patients were persons from IMT, equipment operators, and paramedics. The 79 case-patients were deployed from 17 states. Of 63 crews, 26 (41.2%) had >1 SARS-CoV-2–positive responder. Case-patients were primarily men (83.5%); median age was 39 years (range 20–66 years). Twenty-four (30.4%) case-patients identified as non-Hispanic, 21 (26.6%) identified as Hispanic or Latino, and 34 (43.0%) did not disclose ethnicity. Race was unknown for 34 case-patients (43.0%); 28 (35.4%) were White, 12 (15.2%) reported other race, 4 (5.1%) were Black or African American, and 1 (1.3%) was Native American or other Pacific Islander. A total of 41 (51.9%) case-patients reported symptoms; 4 (5.1%) reported no symptoms, and symptom information was unavailable for 34 (43.0%). Thirteen (16.5%) visited an emergency department, 3 (3.8%) were hospitalized, and no deaths were reported.

Among the 79 case-patients, LCDHE completed interviews with 64 (81.0%). During interviews, these 64 responders identified 273 close contacts who were contacted by LCDHE and instructed to quarantine; however, responders often were unable to provide specific locations of their camps and were unable or unwilling to provide names of their close contacts. Therefore, in addition to routine outbreak case investigation, LCDHE worked closely with the IMT’s COVID-19 liaisons for contact tracing. The COVID-19 liaisons provided documentation of responders’ crew assignments, which proved to be a more effective method of contact tracing among responders than asking case-patients to identify close contacts during interviews. Because each crew traveled and camped together, once a case-patient was identified, their entire crew was considered to be close contacts and exposed.

Forty (51%) of the 79 SARS-CoV-2–positive specimens were available for WGS. We obtained high-quality sequences for 24 specimens, of which 21 were collected during September 1–11, 2020, capturing sequencing data for 87.5% (21/24) of the samples available from the first 3 weeks of the outbreak. In all, we identified 8 lineages (B.1, B.1.2, B.1.240, B.1.243, B.1.403, B.1.564, B.1.595, and B.1.1.304). Lineages identified near the end of the outbreak (B.1.204, B.1.243, and B.1.1.304) were not represented in samples sequenced earlier in the outbreak (B.1., B.1.2, B.1.403, B.1.564, B.1.595). Two lineages were present in >1 crew; for example, lineage B.1.403 was observed in 4 crews and lineage B.1.2 was observed in 3. Three samples were assigned to lineage B.1.2 but showed divergent nucleotide sequences, suggesting 3 separate introductions of this lineage. In addition, >1 lineage was identified in 3 crews (Figure 1). For example, lineages B.1.403 and B.1.2 were both present in crew B.

We performed contextual phylogenetic analysis to determine whether interstate or intrastate transmission occurred. We constructed a full contextual tree by using 717 contextual sequences subsampled from the GISAID repository (https://www.gisaid.org), an additional 37 Colorado sequences sequenced at the CDPHE State Public Health Laboratory, and the 24 Cameron Peak Fire consensus sequences (Figure 2, panel A). The analysis revealed 4 clades that provided evidence of possible intrastate and interstate transmission (Figure 2, panels B–E). Twelve Cameron Peak Fire sequences formed a monophyletic clade with sequences collected from 2 Colorado counties with high support values (ultrafast bootstrap support >95% for nodes; Figure 2, panel B). Another sequence from a Cameron Peak Fire responder formed a clade with sequences collected from 3 Colorado counties and additional sequences collected from outside of Colorado but with low support values (ultrafast bootstrap support <95% for nodes; Figure 2, panel C). In addition, in 2 cases, sequences from 2 different responders formed a clade with contextual sequences collected from their state of deployment; 1 clade was supported with high support values but the other was not (Figure 2, panels D and E). Although not all clades were supported with high bootstrap values, low support values might be expected if sequence diversity is insufficient, which could result from either low diversity of SARS-CoV-2 circulating in the United States at the time, or low diversity among samples that were able to be sequenced and deposited in public repositories. Short branch lengths as observed on the tree are indicative of low divergence among sequences (*12*).

**Figure 4:**
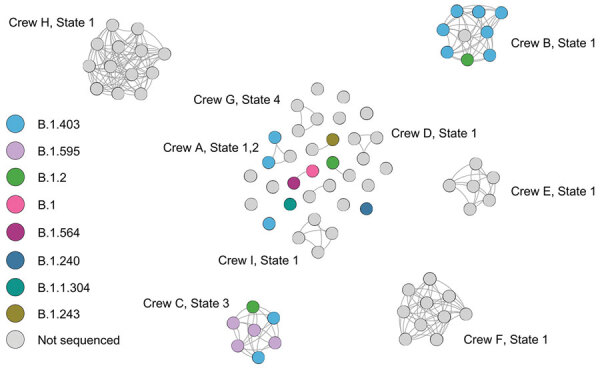
Social network analysis of Cameron Peak firefighter crews with COVID-19, Colorado, USA, August–December 2020. All responders testing positive for SARS-CoV-2 (nodes) are included in this figure to show contact within crews (edges). Crews with >3 firefighters positive with SARS-CoV-2 are labeled.

Social network analysis showed the 79 responders with COVID-19 clustered into 26 crews deploying from 17 states (Figure 4). Nine crews with responders from 10 states experienced >3 cases. We observed multiple lineages within single crews, suggesting multiple points of introduction, probable crew intermingling, and possible lapses in prevention measures such as social distancing.

## Discussion

The Cameron Peak Fire was the largest recorded wildfire in Colorado’s history, burning 208,913 acres. A total of 79 cases of COVID-19 were identified among Cameron Peak Fire responders deployed from 17 states. Multiple points of SARS-CoV-2 introduction were likely because of frequent crew turnover as the wildfire grew, as suggested by WGS and social network analysis results.

Balancing management of a large-scale wildfire and control of COVID-19 among responders created several challenges for disease prevention and mitigation. Frequent responder turnover because of 2- to 3-week deployments, combined with the length of the fire event, resulted in continuous opportunities for introduction of COVID-19 into wildfire camps (*13*). COVID-19 testing was available for incoming responders, but no testing or quarantine was required upon arrival, and no surveillance testing was required during the deployment period. In addition, turnover of responders resulted in several instances in which case-patients in isolation or contacts in quarantine were demobilized back to their home states or deployed to other wildfire responses before case investigation and contact tracing could be completed. In these situations, CDPHE notified the states to which responders were demobilized, and LCDHE coordinated with Cameron Peak IMT to ensure these responders were immediately notified and given instructions to proceed home immediately, avoiding contact with others and stops in indoor public settings during their travel. However, the potential for multistate spread was a major concern when responders were demobilized and sent home or to other responses.

The difficulty of screening responders for COVID-19 symptoms was compounded by challenges differentiating the effects of smoke and high altitude from symptoms of COVID-19. Smoke inhalation can cause several respiratory symptoms that are similar to COVID-19, including coughing, shortness of breath, sore throat, and chest pain (*14*). Altitude sickness symptoms also overlap with COVID-19 symptoms and can include headaches, fatigue, nausea, and vomiting, as well as, in more severe cases, shortness of breath, weakness, and cough (*15*). Symptoms of acute and chronic smoke exposure overlap with and can worsen COVID-19 symptoms, complicating symptom-based identification of COVID-19 (*13*,*16*). Elevations in the fire-affected area ranged from ≈5,200 feet to >10,000 feet, resulting in the potential for altitude sickness for crews, particularly those coming to Colorado from states at lower elevations. 

Often, responders continued to work while they were symptomatic and infectious and did not report symptoms until their illness became severe or they experienced a distinguishing symptom, such as loss of taste or smell. COVID-19 mitigation was further challenged by how fire camps were set up, potentially increasing exposure opportunities. Crews often camped together or worked geographically closely before implementation of mitigation and quarantine measures, potentially increasing exposure opportunities. Furthermore, because these camps were often located in areas with limited cell service, Wi-Fi hotspots provided relatively small areas where responders could access Wi-Fi, creating additional opportunities for exposure when responders gathered closely together in areas where Wi-Fi was available (*9*). Other barriers to the public health response included some responders’ distrust of their positive SARS-CoV-2 test results because of lack of symptoms or overlap with smoke inhalation symptoms. Further, many responders were employed as contractors and were not provided paid sick leave to cover quarantine or isolation. Fire response coordinators and commanders indicated that some crew members might have been hesitant to report symptoms or get tested because of concerns over having to quarantine or isolate without pay. Challenges in gathering complete symptom information could be caused by responders’ reluctance to be pulled from their crew, which could further straining resources during the response. Contact tracing was challenging early in the investigation because case-patients were unable to identify their close contacts or unwilling to provide names of close contacts to avoid quarantine. Further, responders and response commanders were resistant to implementing full quarantines because staffing needs were strained by the severity of the Cameron Peak Fire and other wildfires happening concurrently in the region. Critical infrastructure-modified quarantine and testing-based strategies were used when full quarantines were not feasible, including release from quarantine after a negative RT-PCR result from a specimen collected 7 days after exposure (which was not a recommended practice under standard quarantine guidance at that time) or monitoring responders for symptoms while allowing them to continue working during quarantine (*17*).

The results of WGS and social network analysis suggest multiple SARS-CoV-2 introduction events throughout the wildfire response, as well as spread both between and within crews. The presence of sequences from a single lineage in >1 crew combined with near-identical nucleotide changes observed among these sequences suggest intercrew transmission or transmission between fire crews and nearby communities (Figure 2, panel B). Contextual analysis suggests possible transmission events linked to Cameron Peak Fire responders from both outside and within the state of Colorado; in a few instances, analysis suggested transmission from the state from which an individual was deployed and in other instances from surrounding counties within the state of Colorado. One state deployment introduction (state 5) and 1 Colorado county introduction (Colorado county A) are well supported by bootstrapping, but in the other 2 instances, support was weak. This result might be the result of low sequence diversity across many states present in sequences available in public repositories from this time period.

The first limitation of our study is that COVID-19 cases were likely underreported because of insufficient testing and lack of reporting of symptoms by responders. Surveillance testing was optional and the overlap between COVID-19 symptoms and symptoms associated with smoke inhalation and altitude sickness might have led some persons not to get tested when symptomatic. Second, only 51% of outbreak-related specimens were available for WGS because not all specimens were sent to the CDPHE laboratory, including those collected through the local hospital; therefore, results might not be complete. Finally, social network epidemiologic links were assumed for responders on the same crew but lacked more robust data showing intercrew mingling during and outside of response activities.

Many lessons were learned in this COVID-19 outbreak during a wildfire response. Open communication between fire response agencies and public health agencies enabled enhanced prevention strategies. Fire response agencies should consider symptom screening and testing of all arriving responders to limit introduction of SARS-CoV-2 into fire camps; educating responders about potentially overlapping symptoms of smoke inhalation, COVID-19, and altitude (when relevant); and improving physical distancing of crews onsite. Surveillance testing offers the ability to detect cases early and to prevent transmission before an outbreak occurs (*18*). Rapid testing options, such as the use of rapid antigen tests, can provide many benefits in wildfire response and other emergency management settings, including quick turnaround of results, which can minimize the need to quarantine critical responders while awaiting results; encouraging action in response to mild symptoms that might otherwise be dismissed as the result of smoke or altitude, because it is a quick and easy option to differentiate symptoms; and ease of implementation in remote and nonmedical settings, not requiring transport of persons off-site or coordination with nearby medical facilities. Response agencies should work with jurisdictional public health agencies at the beginning of each response to determine what testing options are currently available and how best to implement testing of responders. Rapidly identifying cases would lead to timely case investigations and contact tracing activities that could help mitigate spread of disease by enabling timely isolation of case-patients and quarantine of close contacts. Policies to compensate responders for time spent in isolation or quarantine could improve compliance with testing and screening procedures. During the response, fire response agencies recommended mask use, especially when other social distancing measures were difficult to maintain. Continuing the use of masks in indoor settings or close interactions with others could be considered in areas of high transmission even in the absence of local public health requirements. In current and future fire seasons, We encourage COVID-19 vaccination and surveillance testing, particularly given the challenges of implementing other mitigation techniques in resource-constrained fire responses. Response agencies should consider collaborating with public health agencies to ensure that appropriate disease control measures are put in place when COVID-19 has been identified among responders, including encouraging cooperation of persons who are identified as case-patients or close contacts to prevent the spread of disease. The lessons learned during this outbreak can contribute to developing best practices for managing wildfire response and outbreaks of COVID-19 and other communicable diseases among responders to large-scale emergency events.

Appendix Additional information about investigation of COVID-19 outbreak among wildland firefighters during wildfire response, Colorado, USA, 2020
