# Supplementary material for: Investigation of COVID-19 Outbreak among Wildland Firefighters during Wildfire Response, Colorado, USA, 2020
Source: Emerg Infect Dis. 2022 Aug;28(8):1551–8. doi: 10.3201/eid2808.220310 (PMC9328929; doi:10.3201/eid2808.220310)
Supplement: Appendix — Additional information about investigation of COVID-19 outbreak among wildland firefighters during wildfire response, Colorado, USA, 2020 [file 22-0310-Techapp-s1.pdf]

Article DOI: https://doi.org/10.3201/eid2808.220310

Investigation of COVID-19 Outbreak among Wildland Firefighters during Wildfire Response, Colorado, USA, 2020

Appendix

Sequencing and Sequence Assembly

RNA was isolated from nasopharyngeal swab specimens using the MagMAX Viral/Pathogen Nucleic Acid Isolation Kit (Thermo Scientific, Waltham, MA, USA) with the 200 µL sample input volume automated method using the KingFisher Flex Magnetic Particle Processor (Thermo Scientific, Waltham, MA, USA) in accordance with the TaqPath COVID-19 Combo Kit (Thermo Scientific, Waltham, MA, USA) (*1*). Samples were then screened with PCR for sequencing viability using the TaqPath COVID-19 Combo Kit PCR assay (Thermo Scientific, Waltham, MA, USA). Specimens were then sequenced on either Illumina MiSeq (Illumina, San Diego, California, USA) or Oxford Nanopore Technology (ONT) GridION (Oxford Nanopore Technologies, Oxford, UK) sequencing platforms with slight modifications following the ARTIC V3 tiled PCR amplicon sequencing protocol for Illumina (*2*) and ONT (*3*) respectively for cDNA preparation and tiled PCR amplification using the ARTIC V3 primer set. For specimens sequenced on Illumina MiSeq, we used the Illumina DNA Prep Kit (Illumia, San Diego, California, USA) for library preparation. For specimens sequenced on ONT GridION, we followed the ARTIC V3 protocol native barcoding method for library preparation. For Illumina MiSeq we used 150 bp paired-end reads and for ONT we used 400–700 bp reads. Each sequencing plate contained a non-template negative control.

We performed reference based viral genome assembly for both Illumina data and ONT gridiron data. For Illumina data we performed a reference based viral genome assembly in two parts. First we used the Monroe pipeline for the alignment of sequencing reads to the reference genome (https://staphb.org/staphb_toolkit/workflow_docs/monroe/; https://github.com/StaPH-B/staphb_toolkit). We then generated the consensus genome using the *consensus* function from

the package iVar (https://github.com/andersen-lab/ivar) (*4*), using a minimum allele frequency of 0.6, a minimum sequencing depth of 10x and a minimum base quality of 30. For ONT GridION data, we performed reference based viral genome assembly using a custom workflow based on the ARTIC bioinformatics nanopore protocol (https://artic.network/ncov-2019/ncov2019-bioinformatics-sop.html) and implemented on the Terra.bio cloud computer platform. Briefly, we filtered reads by length and removed low quality reads using guppy plex. Primer sequences were removed from reads, reads were mapped to the reference genome, and consensus genome sequences were generated using medaka as implemented in the ARTIC bioinformatics protocol.

For both Illumina and ONT data, we assessed the average sequencing depth and percent genome coverage of each consensus genome using custom python scripts (https://github.com/CDPHE/python_scripts_for_terra_workflows). We removed insertions not previously documented in the genome as these were likely due to sequencing and/or assembly error using custom python scripts (https://github.com/CDPHE/sars-cov-2_indel_finder). We defined high quality assembled genomes as those with at least 90 percent coverage across the reference genome. In cases where we could not obtain high quality sequences from the specimen, it could have been due to low viral titer at the time of specimen collection resulting in low RT-PCR Ct values and/or specimen degradation.

All high quality assembled consensus genomes are published to the GISAID and NCBI Genbank repositories and sequencing read data has been published to the NCBI SRA repository (Appendix Tables 1, 2).

Lineage Characterization and Phylogenetic Analyses

To determine relatedness among the Cameron Peak Fire consensus genome sequences and to infer possible transmission events, we first determined the lineage of each sequence using the software PANGOLIN v3.1.1 (https://github.com/cov-lineages/pangolin). Next we constructed a focal phylogenetic tree using the Cameron Peak Fire consensus genome sequences. We aligned consensus genomes using MAFFT v7.471 (*5*) and constructed a maximum likelihood tree using IQ-Tree v.1.6.1 (http://www.iqtree.org) using 1000 ultrafast bootstrap replicates. We visualized the phylogeny using the Python library ete3 v3.1.2 (http://etetoolkit.org/) and custom python scripts.

To investigate the potential for multistate lineage introduction and/or community transmission, we constructed a contextual phylogenetic tree. We first downloaded sequences from GISAID meeting the following criteria: 1) samples collected from human hosts, 2) sequences with complete coverage, 3) samples collected within the United States with locality information to at least the level of state, and 4) samples collected between August 15, 2020 and December 21, 2020, corresponding to 2 weeks before and 2 weeks following the Cameron Peak Fire outbreak (data downloaded July 7, 2021). This resulted in 44,814 sequences. To these sequences we added an additional 872 sequences from samples collected in Colorado between August 15,2020 and December 21, 2020 that were sequenced at the CDPHE State Public Health Laboratory and for which we had county level collection localities. This resulted in a total of 45,686 contextual sequences. Next to determine which sequences among our contextual sequences were most genetically similar to our focal Cameron Peak Fire sequences, we used the *priorities.py* python script (*6*) from the NEXTSTRAIN workflow for SARS-CoV-2 (https://github.com/nextstrain/ncov). This script ranks sequences in order of their similarity to the set of focal sequences and is used as input for the AUGUR *filter* subcommand (https://github.com/nextstrain/augur). Next we filtered the contextual sequences using the *filter* subcommand from AUGUR v.12.0.0. We set the subsampling parameters to group samples by collection date and subsample a max of 1,000 sequences and set the minimum sequence length to 27,000 bp. This subsampling resulted in a total of 778 sequences (717 sequences from GISAID, 37 additional Colorado sequences sequenced at CDPHE State Public Health Laboratory, and 24 focal Cameron Peak Fire sequences) used to build our contextual phylogenetic tree. We aligned the sequences using MAFFT v7.471 and constructed a maximum likelihood tree using IQ-Tree v.1.6.1 using 1000 ultrafast bootstrap replicates. We visualized the phylogeny using the Python library ete3 v3.1.2 and custom python scripts. To help with visualization we pruned the final contextual phylogenetic tree to 200 sequences, while maintaining sequences forming monophyletic groups with the 24 Cameron Peak Fire sequences.

Additional Information about Figure 3 (https://wwwnc.cdc.gov/EID/article/28/8/22-0310-F3.htm)

The 24 Cameron Peak firefighter sequences served as focal sequences and a total of 45,686 sequences served as contextual sequences: 44,814 sequences downloaded from the

GISAID repository (*7*) collected from human specimens testing positive for SARS-CoV-2 within the United States during August 15–December 21, 2020 (representing 1 week before and 1 week after the first and last reported positive cases, respectively) and 872 sequences from human specimens collected from Colorado during the same period and sequenced internally at the Colorado Department of Public Health and Environment state laboratory. Next, all contextual sequences were ranked based on their sequence similarity to the 24 Cameron Peak sequences using Nextstrain's ncov priorities python script (*8*). Contextual sequences were then filtered using Augur's (Version 10.2.0) *filter* subcommand with parameters specified to group sequences by collection date and subsample a maximum of 1,000 sequences. The final sequence dataset for phylogenetic inference consisted of 754 contextual sequences and the 24 Cameron Peak firefighter focal sequences. Phylogenetic inference was performed using IQTree Version 2.0.3 (http://www.iqtree.org). To access branch support, 5,000 ultrafast bootstrap replicates were used. Phylogenetic tree visualization was performed using the python module ete3 Version 3.1.2 (https://pypi.org/project/ete3).

References

1. Food and Drug Administration. TaqPath COVID-19 Combo Kit Instructions for Use [cited 2022 Apr 14]. https://www.fda.gov/media/136112/download

2. DNA Pipelines R&D. Farr B, Rajan D, Betteridge, Shirley L, Quail M, et al. COVID-19 ARTIC v3 Illumina library construction and sequencing protocol V.5 [cited 2022 Apr 14]. https://www.protocols.io/view/covid-19-artic-v3-illumina-library-construction-an-j8nlke665l5r/v5

3. Quick J. nCoV-2019 sequencing protocol v3 (LoCost) V.3 [cited 2022 Apr 14]. https://www.protocols.io/view/ncov-2019-sequencing-protocol-v3-locost-bh42j8ye

4. Grubaugh ND, Gangavarapu K, Quick J, Matteson NL, De Jesus JG, Main BJ, et al. An amplicon-based sequencing framework for accurately measuring intrahost virus diversity using PrimalSeq and iVar. Genome Biol. 2019;20:8. PubMed https://doi.org/10.1186/s13059-018-1618-7

5. Katoh K, Misawa K, Kuma K, Miyata T. MAFFT: a novel method for rapid multiple sequence alignment based on fast Fourier transform. Nucleic Acids Res. 2002;30:3059–66. PubMed https://doi.org/10.1093/nar/gkf436

6. Metz AR, Bauer M, Epperly C, Stringer G, Marshall KE, Webb LM, et al. CDPHE/outbreak_investigation_of_COVID-19_among_wildland_firefighters [cited 2022 Apr 12]. https://github.com/CDPHE/Outbreak_Investigation_of_COVID-19_Among_Wildland_Firefighters

7. Elbe S, Buckland-Merrett G. Data, disease and diplomacy: GISAID's innovative contribution to global health. Glob Chall. 2017;1:33–46. PubMed https://doi.org/10.1002/gch2.1018

8. Nextstrain. Ncov: GitHub [cited 2021 Aug 19]. https://github.com/nextstrain/ncov

Appendix Table 1. Accession IDs of 24 high-quality Cameron Peak Fire sequences, Colorado, USA*

Sequence Name	NCBI BioProject Accession	NCBI BioSample Accession	NCBI SRA Accession	NCBI GenBank Accession	GISAID Accession
Crew A sequences					
hCoV-19/USA/CO-CDPHE-2009021317/2020	PRJNA686984	SAMN17130086	SRR13374028	MW645995	EPI_ISL_677638
hCoV-19/USA/CO-CDPHE-2009021339/2020	PRJNA686984	SAMN17130087	SRR13374027	MW645996	EPI_ISL_677639
Crew B sequences					
hCoV-19/USA/CO-CDPHE-2009042396/2020	PRJNA686984	SAMN17130092	SRR13374021	MW645824	EPI_ISL_710222
hCoV-19/USA/CO-CDPHE-2009042404/2020	PRJNA686984	SAMN17130093	SRR13374020	MW645920	EPI_ISL_710331
hCoV-19/USA/CO-CDPHE-2009042416/2020	PRJNA686984	SAMN17130094	SRR13374019	MW645921	EPI_ISL_710283
hCoV-19/USA/CO-CDPHE-2009042507/2020	PRJNA686984	SAMN17130095	SRR13374018	MW645998	EPI_ISL_677641
hCoV-19/USA/CO-CDPHE-2009042555/2020	PRJNA686984	SAMN17130096	SRR13374017	MW645999	EPI_ISL_677642
hCoV-19/USA/CO-CDPHE-2009042743/2020	PRJNA686984	SAMN17130097	SRR13374016	MW645723	EPI_ISL_677313
hCoV-19/USA/CO-CDPHE-2009090328/2020	PRJNA686984	SAMN17130123	SRR13373986	MW646003	EPI_ISL_677646
hCoV-19/USA/CO-CDPHE-2009090330/2020	PRJNA686984	SAMN17130124	SRR13373985	MW646004	EPI_ISL_677647
Crew C sequences					
hCoV-19/USA/CO-CDPHE-2009042326/2020	PRJNA686984	SAMN17130091	SRR13374022	MW645722	EPI_ISL_677312
hCoV-19/USA/CO-CDPHE-2009090308/2020	PRJNA686984	SAMN17130117	SRR13373993	MW645733	EPI_ISL_677270
hCoV-19/USA/CO-CDPHE-2009090312/2020	PRJNA686984	SAMN17130118	SRR13373992	MW645734	EPI_ISL_677281
hCoV-19/USA/CO-CDPHE-2009090314/2020	PRJNA686984	SAMN17130119	SRR13373991	MW646000	EPI_ISL_677643
hCoV-19/USA/CO-CDPHE-2009090318/2020	PRJNA686984	SAMN17130120	SRR13373990	MW645735	EPI_ISL_677314
hCoV-19/USA/CO-CDPHE-2009090320/2020	PRJNA686984	SAMN17130121	SRR13373988	MW646001	EPI_ISL_677644
hCoV-19/USA/CO-CDPHE-2009090338/2020	PRJNA686984	SAMN17130125	SRR13373984	MW645736	EPI_ISL_677283
Crew I sequences					
hCoV-19/USA/CO-CDPHE-2100078312/2020	PRJNA686984	SAMN23283207	SRR17018872	OL678784	EPI_ISL_6581850
Crew J sequences					
hCoV-19/USA/CO-CDPHE-2009031031/2020	PRJNA686984	SAMN17130088	SRR13374026	MW645997	EPI_ISL_677640
Crew K sequences					
hCoV-19/USA/CO-CDPHE-2009044496/2020	PRJNA686984	SAMN17130098	SRR13374015	MW645724	EPI_ISL_677271
Crew L sequences					
hCoV-19/USA/CO-CDPHE-2009121107/2020	PRJNA686984	SAMN17130126	SRR13373983	MW645737	EPI_ISL_677262
hCoV-19/USA/CO-CDPHE-2009121110/2020	PRJNA686984	SAMN17130127	SRR13373982	MW645738	EPI_ISL_677279
Crew M sequences					
hCoV-19/USA/CO-CDPHE-2100042143/2020	PRJNA686984	SAMN19411787	SRR17435600	OM249427	EPI_ISL_2309210
Crew N sequences					
hCoV-19/USA/CO-CDPHE-2010022040/2020	PRJNA686984	SAMN19411784	SRR17435859	OM249281	EPI_ISL_2309207

*Sequences listed by crew as referred to in Figure 2 (https://wwwnc.cdc.gov/EID/article/28/8/22-0310-F2.htm).

Appendix Table 2. Accession IDs of additional Colorado sequences sequenced at CDPHE State Public Health Laboratory*

Sequence Name	NCBI BioProject Accession	NCBI BioSample Accession	NCBI SRA Accession	NCBI GenBank Accession	GISAID Accession
County A sequences					
hCoV-19/USA/CO-CDPHE-2009170340/2020	PRJNA686984	SAMN17130186	SRR13373917	MW645951	EPI_ISL_710345
hCoV-19/USA/CO-CDPHE-2009170292/2020	PRJNA686984	SAMN17130181	SRR13373922	MW645946	EPI_ISL_710342
hCoV-19/USA/CO-CDPHE-2009163553/2020	PRJNA686984	SAMN17130143	SRR13373964	MW646006	EPI_ISL_677649
hCoV-19/USA/CO-CDPHE-2009164054/2020	PRJNA686984	SAMN17130161	SRR13373944	MW645928	EPI_ISL_710333
hCoV-19/USA/CO-CDPHE-2008161331/2020	PRJNA686984	SAMN17130051	SRR13374066	MW645817	EPI_ISL_710217
hCoV-19/USA/CO-CDPHE-2009170150/2020	PRJNA686984	SAMN17130176	SRR13373928	MW645943	EPI_ISL_710341
hCoV-19/USA/CO-CDPHE-2009170084/2020	PRJNA686984	SAMN17130172	SRR13373932	MW645939	EPI_ISL_710339
hCoV-19/USA/CO-CDPHE-2100008733/2020	PRJNA686984	SAMN17250951	SRR13404609	MW645561	EPI_ISL_771183
County B Sequences					
hCoV-19/USA/CO-CDPHE-2008170075/2020	PRJNA686984	SAMN17130056	SRR13374061	MW645710	EPI_ISL_677304
hCoV-19/USA/CO-CDPHE-2100060009/2020	PRJNA686984	SAMN17903010	SRR13703951	MW629399	EPI_ISL_983861
hCoV-19/USA/CO-CDPHE-2011221706/2020	PRJNA686984	SAMN18173874	SRR13867969	MW715242	EPI_ISL_1169713
hCoV-19/USA/CO-CDPHE-2100060046/2020	PRJNA686984	SAMN17903011	SRR13703950	MW629400	EPI_ISL_983862
hCoV-19/USA/CO-CDPHE-2011134677/2020	PRJNA686984	SAMN19413147	SRR17436226	OM171064	EPI_ISL_2310376
hCoV-19/USA/CO-CDPHE-2011134720/2020	PRJNA686984	SAMN19413148	SRR17436234	OM170758	EPI_ISL_2310377
hCoV-19/USA/CO-CDPHE-2100117646/2020	PRJNA686984	SAMN19413606	SRR17489341	OM211796	EPI_ISL_2310835
hCoV-19/USA/CO-CDPHE-2100106281/2020	PRJNA686984	SAMN19413558	SRR17489350	OM211622	EPI_ISL_2310787
hCoV-19/USA/CO-CDPHE-2100060065/2020	PRJNA686984	SAMN19413181	SRR17436998	OM170407	EPI_ISL_2310410
hCoV-19/USA/CO-CDPHE-2009262446/2020	PRJNA686984	SAMN19407744	SRR17350928	OM067237	EPI_ISL_1540131
hCoV-19/USA/CO-CDPHE-2100145835/2020	PRJNA686984	SAMN19413673	SRR17489016	OM211591	EPI_ISL_2310902
hCoV-19/USA/CO-CDPHE-2010022230/2020	PRJNA686984	SAMN19407745	SRR17350927	OM067433	EPI_ISL_1540132
hCoV-19/USA/CO-CDPHE-2100085221/2020	PRJNA686984	SAMN19413110	SRR17436315	OM170772	EPI_ISL_2310339
County C Sequences					
hCoV-19/USA/CO-CDPHE-2100013339/2020	PRJNA686984	SAMN17250967	SRR13404560	MW645576	EPI_ISL_771299
hCoV-19/USA/CO-CDPHE-2011044565/2020	PRJNA686984	SAMN19411925	SRR17435786	OM248836	EPI_ISL_2309348
County D Sequences					
hCoV-19/USA/CO-CDPHE-2100015283/2020	PRJNA686984	SAMN17250997	SRR13404525	MW645604	EPI_ISL_771179
All Other Colorado Sequences					
hCoV-19/USA/CO-CDPHE-2100116162/2020	PRJNA686984	SAMN20509402	SRR15488467	MZ830216	EPI_ISL_3160167
hCoV-19/USA/CO-CDPHE-2100036174/2020	PRJNA686984	SAMN17251037	SRR13404481	MW645994	EPI_ISL_771175
hCoV-19/USA/CO-CDPHE-2100001047/2020	PRJNA686984	SAMN17250939	SRR13404622	MW645549	EPI_ISL_771283
hCoV-19/USA/CO-CDPHE-2100001027/2020	PRJNA686984	SAMN17250938	SRR13404624	MW645548	EPI_ISL_771282
hCoV-19/USA/CO-CDPHE-2100014442/2020	PRJNA686984	SAMN17250979	SRR13404544	MW645587	EPI_ISL_771305
hCoV-19/USA/CO-CDPHE-2008170097/2020	PRJNA686984	SAMN17130057	SRR13374060	MW645711	EPI_ISL_677305
hCoV-19/USA/CO-CDPHE-2010191275/2020	PRJNA686984	SAMN17130202	SRR13373898	MW645964	EPI_ISL_710354
hCoV-19/USA/CO-CDPHE-2100038259/2020	PRJNA686984	SAMN17251055	SRR13404461	MW645657	EPI_ISL_771350
hCoV-19/USA/CO-CDPHE-2010294547/2020	PRJNA686984	SAMN20794120	SRR15460147	MZ832789	EPI_ISL_3403786
hCoV-19/USA/CO-CDPHE-2100036488/2020	PRJNA686984	SAMN17251043	SRR13404474	MW645645	EPI_ISL_771342
hCoV-19/USA/CO-CDPHE-2011200501/2020	PRJNA686984	SAMN17250909	SRR13404591	MW645519	EPI_ISL_771258
hCoV-19/USA/CO-CDPHE-2011193762/2020	PRJNA686984	SAMN17250899	SRR13404435	MW645509	EPI_ISL_771161
hCoV-19/USA/CO-CDPHE-2100280404/2020	PRJNA686984	SAMN23283277	SRR17019083	OL679077	EPI_ISL_6581924

*Sequences are listed by county as referred to in Figure 3 (https://wwwnc.cdc.gov/EID/article/28/8/22-0310-F3.htm). CDPHE, Colorado Department of Public Health and Environment.
